# Relationship between participant-reported outcomes, residual beta cell function and metabolic parameters in youth with newly diagnosed type 1 diabetes

**DOI:** 10.1007/s00125-026-06798-z

**Published:** 2026-07-17

**Authors:** Peter N. Taylor, Wai-Yee Cheung, Joe Lagorio Price, Charlotte Boughton, Jane Bowen-Morris, Hayley A. Hutchings, Stephanie J. Hanna, Steve Luzio, Danijela Tatovic, Ashish Marwaha, Julia Fuchs, Anna Lam, John W. Gregory, Roman Hovorka, Colin M. Dayan

**Affiliations:** 1 Division of Infection and Immunity, Cardiff University School of Medicine, Cardiff, UK; 2 Department of Immunology and Immunotherapy, University of Birmingham, Birmingham, UK; 3 Diabetes Research Group, Swansea University, Swansea, UK; 4 Wellcome–Medical Research Council Institute of Metabolic Science, University of Cambridge, Cambridge, UK; 5 Swansea Trials Unit, Swansea University Medical School, Swansea, UK; 6 Department of Medical Genetics, University of Calgary, Calgary, AB Canada; 7 Alberta Diabetes Institute, University of Alberta, Edmonton, AB Canada; 8 Department of Paediatrics, Cardiff University School of Medicine, Cardiff, UK

**Keywords:** CLOuD, C-peptide, Hypoglycaemia fear survey, PEDS, PROMs, Quality of life, USTEKID

## Abstract

**Aims/hypothesis:**

Clinical trials of interventions to preserve beta cell function in new-onset type 1 diabetes frequently employ participant-reported outcome measures (PROMs). However, the expected changes in PROMs scores immediately following diagnosis and their association with residual beta cell function, metabolic markers and continuous glucose monitoring (CGM) are unclear.

**Methods:**

Repeated PROMs including Paediatric Quality of Life Inventory diabetes module (PedsQL) and hypoglycaemia fear survey (HFS) were recorded from participants aged 10–18 years with newly diagnosed type 1 diabetes and their parents in two clinical trials: CLOuD (*N*=97, hybrid closed loop [HCL] vs multiple daily injections [MDI]) and USTEKID (*N*=72, ustekinumab immunotherapy vs placebo). Scores were compared with serial mixed meal-stimulated C-peptide levels (AUC C-peptide), HbA_1c_ and CGM data.

**Results:**

PedsQL and HFS scores for children/adolescents and their parents showed wide variation between individuals but did not change substantially within individuals over the first 48 months from diagnosis. Baseline scores were highly predictive of scores at 12–48 months (*p*<0.001). PedsQL scores were higher (better) in those reported by children/adolescents than by their parents (*p*<0.01). In contrast, HFS scores were higher in parents than children (*p*<0.001), indicating more fear. Strong correlations were observed between child and parent scores (*p*<0.001). No significant improvement in these scores was detected following intervention (ustekinumab or HCL). Meta-analysis revealed modest but statistically significant associations between HbA_1c_ and PedsQL (β_(std)_=−0.11; 95% CI −0.20, −0.03) and HFS (β_(std)_=0.11; 95% CI 0.00, 0.21), and between CGM time in range and PedsQL (β_(std)_=0.14; 95% CI 0.03, 0.26) but not HFS (β_(std)_=−0.05; 95% CI −0.16, 0.06). Beta cell function (AUC C-peptide) was strongly associated with HbA_1c_ (β_(std)_=−0.29; 95% CI −0.39, −0.20) and CGM time in range (β_(std)_=0.41; 95% CI 0.30, 0.52). Higher beta cell function showed a trend towards better PedsQL (β_(std)_=0.11; 95% CI −0.03, 0.25) and lower HFS (β_(std)_=−0.05; 95% CI −0.17, 0.07) but this did not reach statistical significance.

**Conclusions/interpretation:**

PedsQL and HFS scores changed little during the first 48 months after diagnosis of type 1 diabetes. These scores showed modest but statistically significant associations with measures of glucose management (HbA_1c_ and CGM time in range), whereas the relationships with residual beta cell function (C-peptide) were weaker and did not reach significance. The modest size of these effects suggests current PROMs capture only limited aspects of the clinical benefit associated with beta cell preservation. Future research should incorporate psychometric instruments that are specifically adapted for young people using modern diabetes technologies and undergoing disease-modifying therapy, to ensure outcomes are meaningfully represented in early-stage type 1 diabetes trials.

**Graphical Abstract:**

**Figure:**
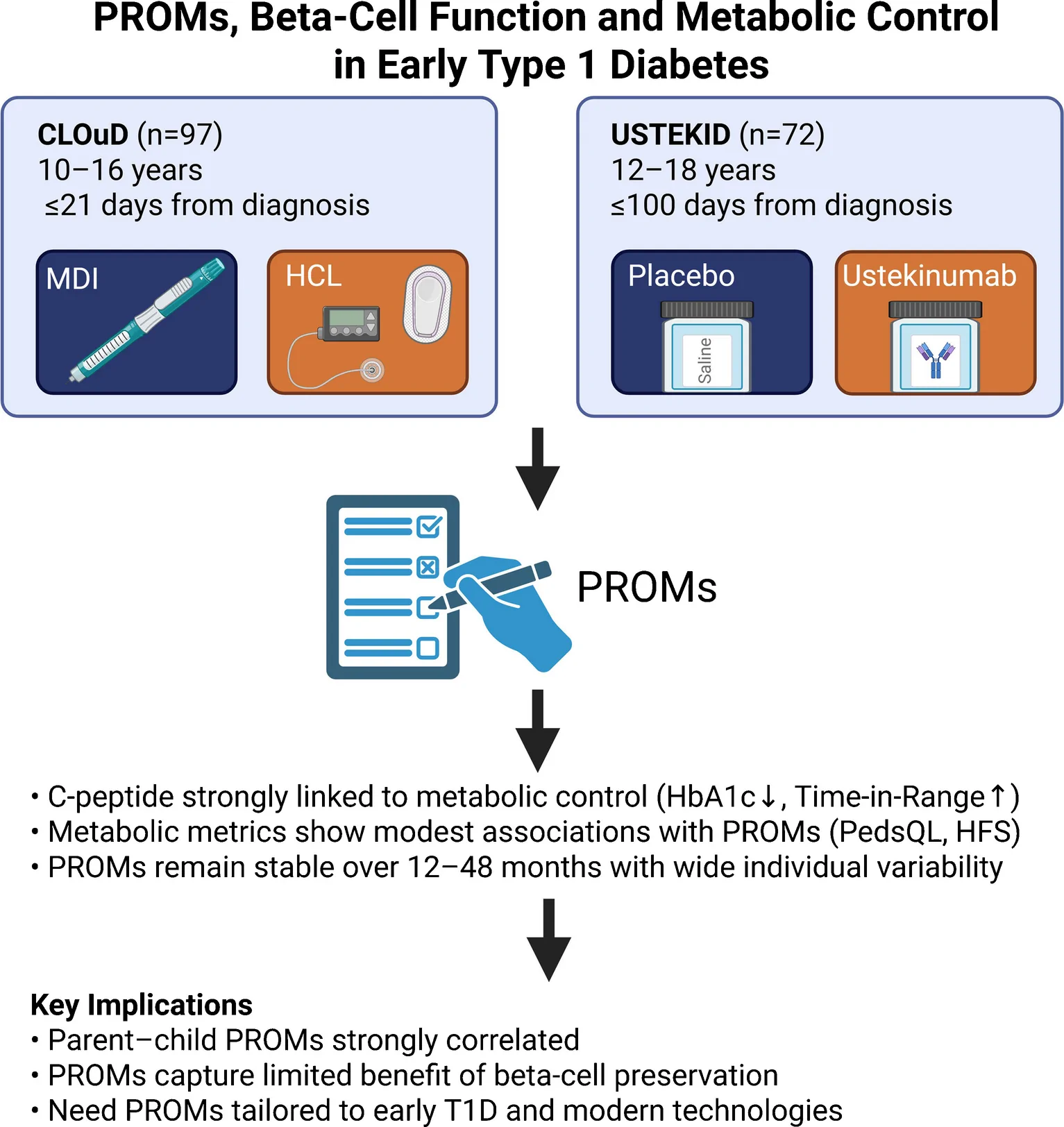

**Supplementary Information:**

The online version contains peer-reviewed but unedited supplementary material available at https://doi.org/10.1007/s00125-026-06798-z.

**Figure:**
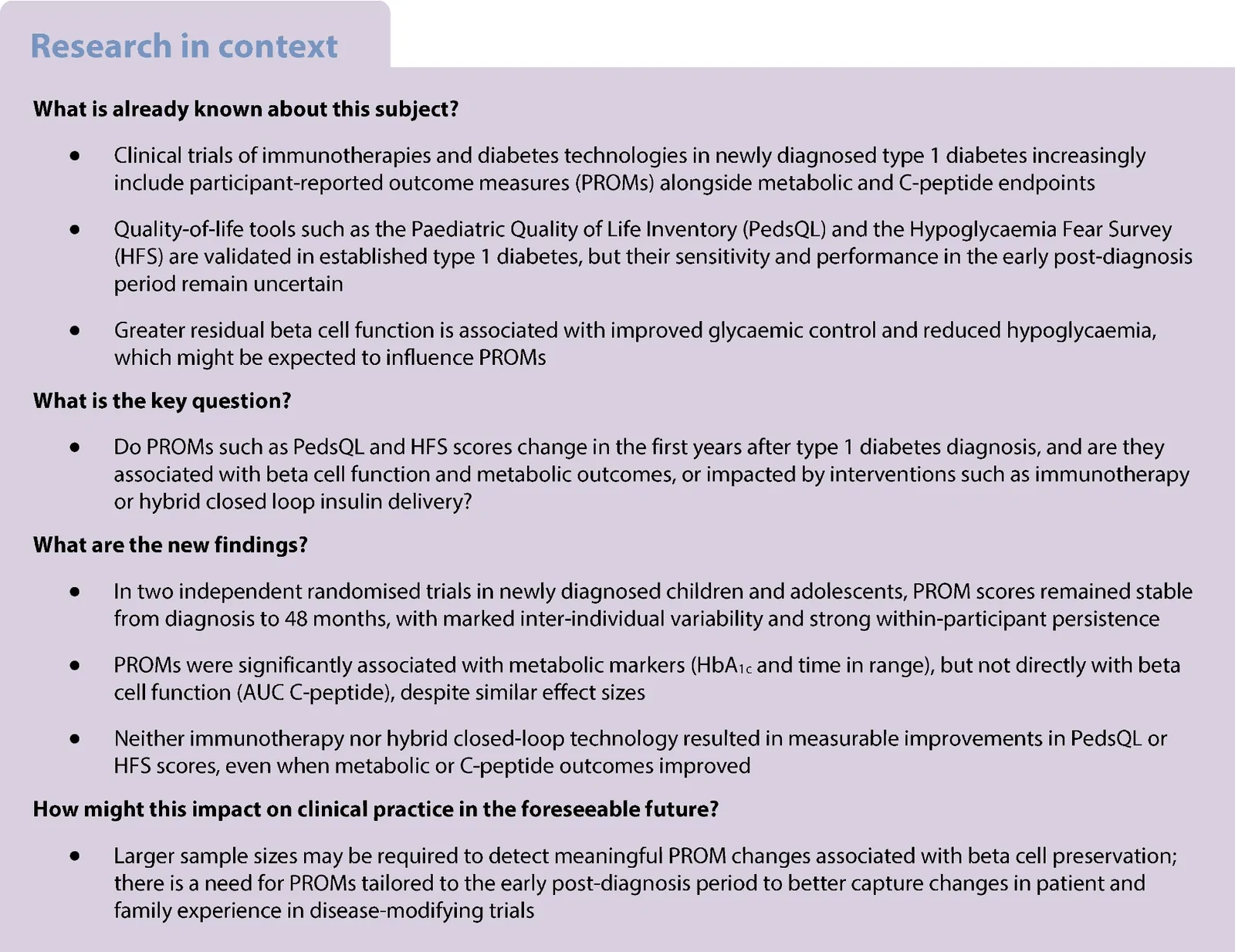

## Introduction

Following the licensing of teplizumab as the first disease process-modifying treatment in type 1 diabetes [1], there has been increased interest in clinical trials of interventions to preserve beta cell function in new-onset type 1 diabetes. These studies have focused on the evaluation of outcomes such as C-peptide and HbA_1c_ [2, 3]. With psychological care becoming more central to participant-centred management [4] and a drive to include outcomes important to participants and families, many recent trials have adopted participant-reported outcome measures (PROMs) as additional endpoints [5]. Recommended core outcome sets in established type 1 diabetes already include diabetes-related distress, treatment satisfaction, severe hypoglycaemia, self-management behaviour and perceived level of management alongside metabolic markers [5]. Several validated instruments exist, targeting general quality of life, diabetes-specific functioning, treatment satisfaction and fear of hypoglycaemia [6–8]. A recent systematic evaluation of PROMs used in paediatric diabetology highlights significant variability in psychometric quality and suitability for modern diabetes care contexts [9].

These tools have shown that individuals with type 1 diabetes often report lower quality of life and higher rates of depression and distress compared with peers without diabetes, particularly when glucose levels are outside recommended targets [10–12]. Even at diagnosis there is a difference between PROMs with diabetes subtypes [13]. It should be noted, however, that these instruments were developed and validated in people with established type 1 diabetes. Their performance in newly diagnosed populations, still adjusting emotionally and practically to the diagnosis, remains unclear. This is important to clarify as their insight into the quality of life impact of type 1 diabetes is limited in the early days following diagnosis.

The relationship between residual beta cell function, PROMs and metabolic management is potentially complex. Higher C-peptide levels (reflecting better beta cell function) are associated with reduced frequency and/or severity of hypoglycaemia [14, 15], which is often a major source of fear and anxiety in people with type 1 diabetes and their families. This may reduce fear of hypoglycaemia independently of overall glucose management but may also improve metabolic management by increasing confidence in aiming for lower blood glucose levels. In addition, improved beta cell function directly promotes better metabolic management by reducing glycaemic variability and postprandial glucose rises, making it easier to achieve targets [3, 16]. However, it should be noted there is the potential for reverse causation in the relationship between metabolic management and PROMs, with more stressed individuals able to concentrate less on managing their glucose levels [17, 18]. Furthermore, advanced insulin delivery systems such as hybrid closed loop (HCL) therapy may improve metabolic management and reduce hypoglycaemia and thereby improve PROMs and HbA_1c_ independently of beta cell function [3, 19–21].

It is therefore unclear whether PROMs as outcomes in studies of interventions to preserve beta cell function will represent indirect proxy measurements of metabolic management or can be impacted independently. Moreover, the longitudinal stability of PROMs scores in early type 1 diabetes, and their relationship to metabolic and beta cell function soon after diagnosis as well as their sensitivity to change, has not been well characterised.

To address these questions, we analysed data from two recent trials in newly diagnosed youth with type 1 diabetes: USTEKID [22] and CLOuD [23]. Both evaluated beta cell preservation as a primary outcome, assessed by AUC C-peptide following a mixed meal tolerance test at 12 months. USTEKID tested ustekinumab vs placebo alongside standard care [22], while CLOuD compared HCL insulin therapy with multiple daily injections (MDI) [20]. Both studies also collected repeated PROMs, metabolic and C-peptide data, enabling us to examine the complex relationships between these domains over time. In addition, both studies used the Paediatric Quality of Life Inventory diabetes module (PedsQL) [7] and Hypoglycaemia fear survey (HFS) assessments [8], making it possible to combine the data for these endpoints for increased statistical power.

## Methods

### Study cohorts

USTEKID was a phase II multicentre, double-blind, randomised placebo-controlled trial of ustekinumab in children and adolescents aged 12–18 years within 100 days of diagnosis of type 1 diabetes. This study has been described in detail previously [22]. Data were broadly typical of UK new-onset type 1 diabetes for age and sex, but detailed socioeconomic information was not reported. Sex was recorded at enrolment as part of the parent trials based on routine clinical documentation (reflecting parent/guardian report and/or medical records at diagnosis). Gender identity was not collected, and sex was included as a covariate in the statistical analyses.

A total of 72 participants were randomised in a 2:1 ratio to ustekinumab and placebo. Participants were followed up for 52 weeks and had regular reviews for beta cell preservation, metabolic and quality-of-life assessments. In USTEKID, participants received standard insulin therapy via MDI with continuous glucose monitoring (CGM) support; automated (AID) or HCL systems were not used in this trial. Participants and their families received routine diabetes education and ongoing clinical support according to standard UK paediatric practice.

The CLOuD trial [23] was a multicentre, open-label, parallel-group, randomised trial comparing HCL delivery and standard insulin therapy (control) for 24 months, with a further extension phase of 24 months with continuation of treatment allocated at randomisation. It recruited 97 youths aged 10–16 years randomised in a 1:1 fashion within 21 days after a diagnosis of type 1 diabetes. This study has been described in detail [23]. Overall, the age range, sex distribution, frequency of diabetic ketoacidosis at presentation, and ethnic distribution are broadly consistent with UK paediatric type 1 diabetes cohorts, although ethnic minority groups remain under-represented relative to the most diverse urban populations.

Training in insulin use, device management and CGM interpretation was provided to all participants and parents before randomisation and reinforced during follow-up visits.

#### Participant- and parent-reported outcome PedsQL measures

In the USTEKID study PedsQL scores consisted of the Paediatric Quality of Life inventory diabetes module (PedsQL) [7] and Hypoglycaemia Fear Survey [8] at baseline, 6 months and 12 months. In CLOuD PedsQL scores consisted of the Paediatric Quality of Life inventory diabetes module [7] and Hypoglycaemia Fear survey [8] at baseline and at 12, 24, 36 and 48 months.

The Paediatric Quality of Life Inventory (PedsQL) 3.2 Diabetes Module [7] assesses diabetes-specific quality of life across five domains: diabetes symptoms, treatment barriers, treatment adherence, worry and communication. Subscale and total scores range from 0 to 100, with higher scores indicating better quality of life. In paediatric diabetes cohorts, mean total scores typically fall around 70 (SD ~15). The Hypoglycaemia Fear Survey (HFS) [8] includes two validated subscales: the Behaviour subscale (HFS-B), assessing behaviours undertaken to avoid hypoglycaemia (e.g. overtreatment, glucose monitoring), and the Worry subscale (HFS-W), assessing cognitive and emotional concerns about hypoglycaemia. Each item is scored from 0 (‘never’) to 4 (‘almost always’), generating mean subscale scores from 0 to 4. Higher scores indicate greater fear of hypoglycaemia; values above approximately 2.5 are generally considered elevated [8]. Both child-report and parent-report versions were used.

Each measure had a parent-specific version which was used for the parent or carer of the participant. All the participant- and parent/carer-reported measures were scored and missing PROMs data handled according to developers’ guidelines [6–8]. Additionally treatment satisfaction was evaluated using the Diabetes Treatment Satisfaction Questionnaire (DTSQ) [6], which assesses satisfaction with diabetes treatment. DTSQ treatment satisfaction scores range from 0 to 36, with higher scores indicating greater treatment satisfaction. Generic psychosocial well-being was additionally assessed using the Strengths and Difficulties Questionnaire (SDQ) [24], a validated measure of emotional symptoms, conduct problems, hyperactivity/inattention, peer relationship problems, and prosocial behaviour. Total difficulties scores range from 0 to 40, with higher scores indicating greater emotional and behavioural difficulties, whereas higher prosocial behaviour scores indicate more positive social functioning.

#### Metabolic outcomes

Metabolic outcomes collected in the trials included secretion of C-peptide, level of HbA_1c_, use of exogenous insulin and insulin-dose-adjusted HbA_1c_ (IDAAC). Secretion of C-peptide was assessed by AUC C-peptide by MMTT, in CLOuD at screening and at 12 months and 24 months, and in USTEKID at screening and at week 28 and week 52 [22, 23]. A blood sample was taken at weeks 0, 12, 28 and 52 for each participant to measure HbA_1c_ in USTEKID [22] and every 3 months throughout the 48 month follow-up in CLOuD [23]. CGM data were also analysed in the 2 weeks prior to clinic visits and the following metrics were recorded: time in range (TIR), SD, time below 3.9 mmol/l (time below range [TBR]) and time above 10.0 mmol/l (time above range [TAR]).

#### Statistical analysis

Participant demographics were summarised by treatment arm for each trial. Continuous variables were standardised where appropriate, and effect sizes are reported per standard deviation (SD). Cross-sectional relationships between metabolic outcomes (C-peptide, HbA_1c_) and PROMs were assessed using ordinary least squares linear regression.

Standardised regression coefficients, β_(std)_, are reported to allow comparison of effect magnitudes across variables with different units. A coefficient represents the expected change (in SD units) in the outcome for each 1 SD increase in the predictor. For instance, a β_(std)_ of 0.14 for the association between TIR and PedsQL indicates that a 1 SD improvement in TIR corresponds to a 0.14 SD higher quality-of-life score.

Differences between participant- and parent-reported outcome measures were evaluated using the Wilcoxon signed rank test. Longitudinal relationships were assessed via mixed-effects models for repeated measures using restricted maximum likelihood estimation. Meta-analyses were conducted by study (CLOuD, USTEKID) and treatment arm (HCL/MDI; ustekinumab/placebo), with estimates pooled using a random-effects model. To account for skewed distributions quantile regression was used to assess quality-of-life outcomes by treatment group. Statistical significance was defined a priori as *p*<0.05. Comparisons between parent and child scores were performed using the paired Wilcoxon signed rank test. All analyses were performed in STATA version 18 (StataCorp, College Station, TX, USA).

#### Ethics

Written informed consent/assent was obtained from all participants. In CLOuD, approval was received from the Cambridge East Research Ethics Committee (16/EE/0286) and the Medicines and Healthcare Products Regulatory Agency [23]. USTEKID was approved by the Wales Research Ethics Committee (REC 3, reference 18/WA/0092) and the UK Medicines and Healthcare products Regulatory Agency [22].

## Results

Data are available for CLOuD (*N*=97) and USTEKID (*N*=72). Details of the study populations are shown in Table 1. The two study populations were very similar although CLOuD participants were generally younger and recruited substantially sooner after diagnosis and with a more even sex balance (Table 1). Peak C-peptide levels were lower in CLOuD than USTEKID, reflecting the earlier age at diagnosis in CLOuD, which is also in keeping with the difference in AUC C-peptide at baseline. Across models, the standardised coefficients were generally in the small-to-moderate range, with most values clustering around 0.10–0.30. According to conventional thresholds, these represent modest associations, indicating that even substantial differences in C-peptide translate into only modest shifts in PROMs, consistent with effect sizes commonly observed in psychosocial and behavioural diabetes research. As expected, higher PedsQL scores indicated better perceived quality of life, and in both trials children and adolescents reported slightly better quality of life than their parents reported on their behalf.

**Table 1:** Baseline characteristics

Characteristic	USTEKID		CLOuD	
	Placebo (*n*=25)	Ustekinumab (*n*=47)	Control (MDI) (*N*=46)	HCL (*N*=51)
Sex				
Male	16 (64)	27 (57)	18 (39)	25 (49)
Female	9 (36)	20 (43)	28 (61)	26 (51)
Age at diagnosis in years				
Mean ± SD	14.28 ± 1.65	13.83 ± 1.74	12 ± 2	12 ± 2
Min, max	12, 18	11, 18	9, 16	10, 16
Age (categorical)				
9–11 years			24 (52)	19 (37)
12–15 years	20 (80)	39 (83)	21 (46)	30 (59)
16–18 years	5 (20)	8 (17)	1 (2)	2 (4)
Age at screening in years				
Mean ± SD	15.0 ± 1.63	14.49 ± 1.78	11.9 ± 1.77	12.2 ± 1.57
Min, max	12.46, 18.77	12.18, 18.53	10, 16	10, 16
Peak C-peptide level at screening (nmol/l)				
0.2–0.7	5 (20)	8 (17)	18 (39)	25 (49)
>0.7	20 (80)	39 (83)	28 (61)	26 (51)
Ethnicity				
White	20 (80)	39 (83)	35 (76)	44 (86)
Mixed race	1 (4)	4 (9)	1 (2)	4 (8)
Black	1 (4)	2 (4)	2 (4)	1 (2)
Asian	1 (4)	1 (2)	1 (2)	2 (4)
Other ethnicity	2 (8)	1 (2)	4 (9)	0

Data are presented as *n* (%) unless stated otherwise

All USTEKID participants were treated with MDI plus CGM; no automated or HCL systems were used

### Analysis by intervention

As previously published, ustekinumab preserved beta cell function at 12 months but there was no significant C-peptide difference by intervention in CLOuD at the primary endpoint [22, 23]. After adjustment for baseline levels, we observed no difference by treatment group at 48 months in CLOuD for PedsQL (*p*=0.40) or HFS (*p*=0.90). No difference was observed for HFS-W (*p*=0.42) and HFS-B (*p*=0.79). In USTEKID we also observed no difference at 12 months for PedsQL (*p*=0.56) or HFS (*p*=0.55). Again, no association was seen in HFS-W subscales (*p*=0.27) and HFS-B (*p*=0.81).

In CLOuD, severe hypoglycaemia events were rare, with five events in three participants in the HCL group and one event in the standard therapy group (incidence 5.4 and 1.2 per 100 person-years, respectively) [23]. In USTEKID [22], no severe hypoglycaemia events were reported during 12 months of follow-up. These low event frequencies did not allow meaningful modelling of within-participant associations between severe hypoglycaemia and HFS scores.

As a result, in the subsequent analyses examining the relationship of beta cell function to PROMs and metabolic outcomes, the intervention and control groups have where appropriate been combined. Note also that cross-sectional analysis at individual time points for each group (HCL/MDI/ustekinumab/placebo) was underpowered to show differences and therefore meta-analysis of both trials across all time points was used.

### Change in PROMs over time since diagnosis and relationship between participant and carer scores

**Fig. 1:**
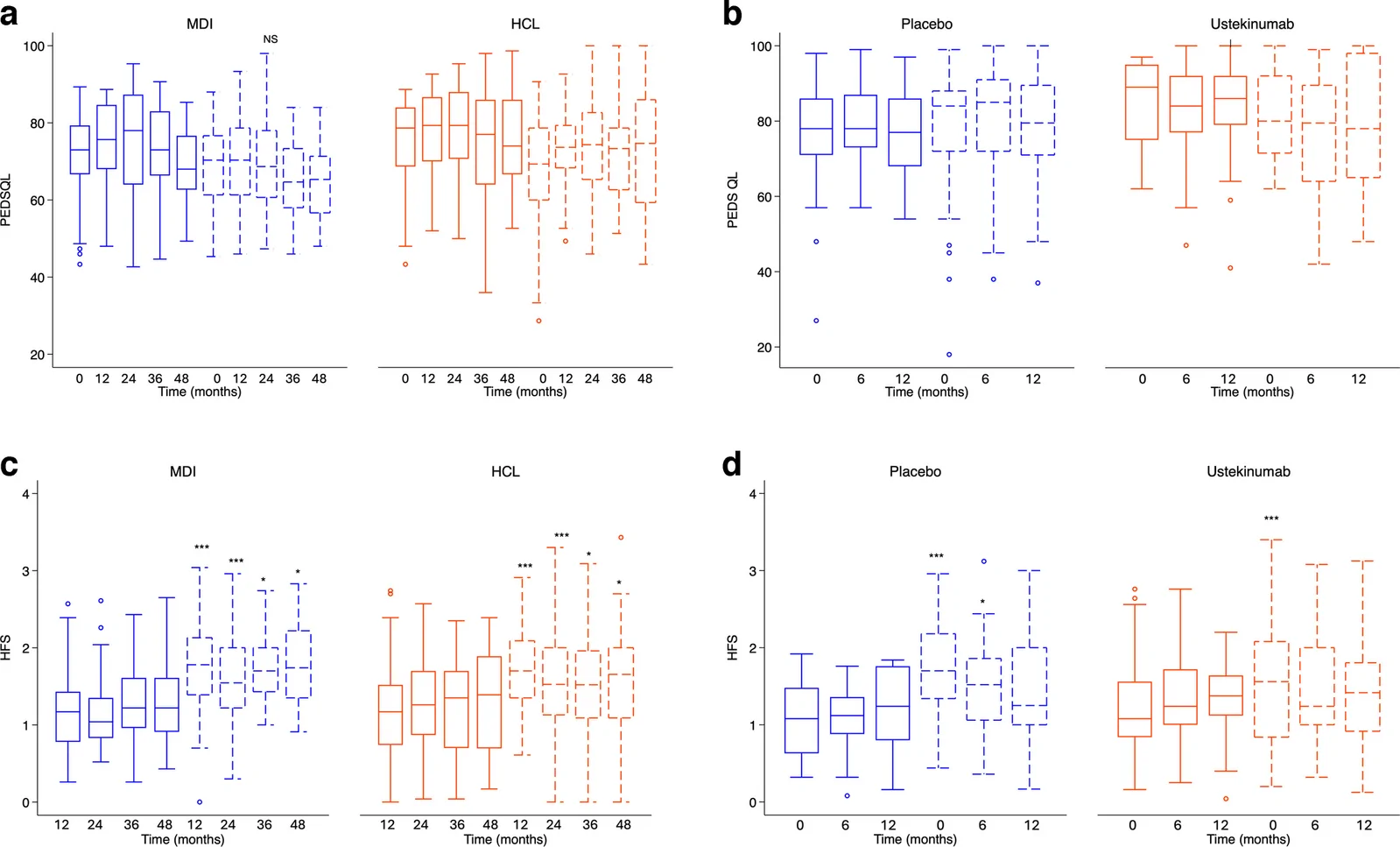
Change in PROMs over time since diagnosis in each study. (**a**) Box plots showing PedsQL scores (median, IQR, 95% range) at 0, 12, 24, 36 and 48 months after diagnosis in participants receiving MDI (blue) and HCL therapy (orange) in the CLOuD study. (**b**) Box plots showing PedsQL scores (median, IQR, 95% range) at 0, 6 and 12 months after diagnosis in participants receiving placebo (blue) and ustekinumab (orange) in the USTEKID study. (**c**) Box plots showing hypofear scores (HFS) (median, IQR, 95% range) at 12, 24, 36 and 48 months after diagnosis in participants receiving MDI (blue) and HCL (orange) in the CLOuD study. (**d**) Box plots showing HFS (median, IQR, 95% range) at 0, 6 and 12 months after diagnosis in participants receiving placebo (blue) and ustekinumab (orange) in the USTEKID study. Solid lines represent participant scores; dashed lines reflect parent/carer scores. *p<0.05, ***p<0.001 level between parent and child scores at this timepoint

We observed there were no significant changes in PedsQL in either participants or parents/carers over the duration of the trial follow-up in the CLOuD (Fig. 1a) and USTEKID studies (Fig. 1b). There were also no significant changes in HFS over the study duration in either study (Fig. 1c, d). There were no significant differences in PedsQL between the participants and the parents; however, parents/carers had consistently higher HFS scores than participants at the majority of time points (Fig. 1c, d).

In the USTEKID study there was no difference over time in participants and parents/carers with regard to DTSQ score (electronic supplementary material [ESM] Fig. 1). In both groups carers had higher treatment satisfaction than their children at baseline and 12 months. In CLOuD there was also no difference over time with regard to SDQ score in participants and parents/carers (ESM Fig. 2); however, in the longer-term follow-up of CLOuD at 3 and 4 years post diagnosis, participants reported higher difficulties than parents/carers (ESM Fig. 2).

Scores at baseline predicted future levels for PedsQL measures. Specifically, baseline PedsQL score predicted 12 month score in USTEKID (β_(std)_=0.64; 95% CI 0.45, 0.83; *p*<0.001; *r*^2^=0.43) (ESM Fig. 3a) and a similar prediction was observed in CLOuD (β_(std)_=0.48; 95% CI 0.25, 0.71; *p*<0.001; *r*^2^=0.22) (ESM Fig. 3b) at 48 months. We also observed a strong association between participants and parents/carers in PedsQL scores at 12 months in USTEKID (β_(std)_=0.38; 95% CI 0.19, 0.57; *p*<0.001; *r*^2^=0.21) and in CLOuD at 48 months (β_(std)_=0.60; 95% CI 0.41, 0.79; *p*<0.001; *r*^2^=0.45).

In CLOuD, across all time points, parents consistently reported higher hypoglycaemia fear scores than their children. The mean total HFS score was 1.68 for parents and 1.23 for children (*p*<0.001), with significant differences also observed for both the HFS-B and HFS-W subscales. Mean HFS-B scores were 1.81 in parents and 1.61 in children (*p*<0.001), while mean worry HFS-W were 1.57 and 0.93, respectively (*p*<0.001). Parent and child scores were moderately correlated within families, with correlation coefficients of *r*=0.33 for HFS-B (*p*<0.001), *r*=0.33 for HFS-W (*p*<0.001) and *r*=0.41 for total HFS (*p*<0.001). These findings indicate that although parents generally experience greater fear of hypoglycaemia than their children, there is a moderate degree of concordance between family members, suggesting shared perceptions and emotional responses related to hypoglycaemia risk.

To aid interpretation, these PROMs values were compared with published reference data. Child-reported PedsQL Diabetes Module scores in our cohort (72 ± 14) and parent-reported scores (78 ± 13) are consistent with established norms from paediatric type 1 diabetes cohorts, where mean scores typically are around 70 (SD 12–15) [7]. HFS-W and HFS-B subscale means in our study also fall within expected ranges described in psychometric validation studies (approximately 1.3–2.0), with values above 2.5 generally considered elevated [8]. This comparison indicates that participants’ perceived quality of life and hypoglycaemia-related concerns were typical for young people early after diagnosis.

### Relationship between AUC C-peptide and metabolic parameters (HbA_1c_, CGM)

**Fig. 2:**
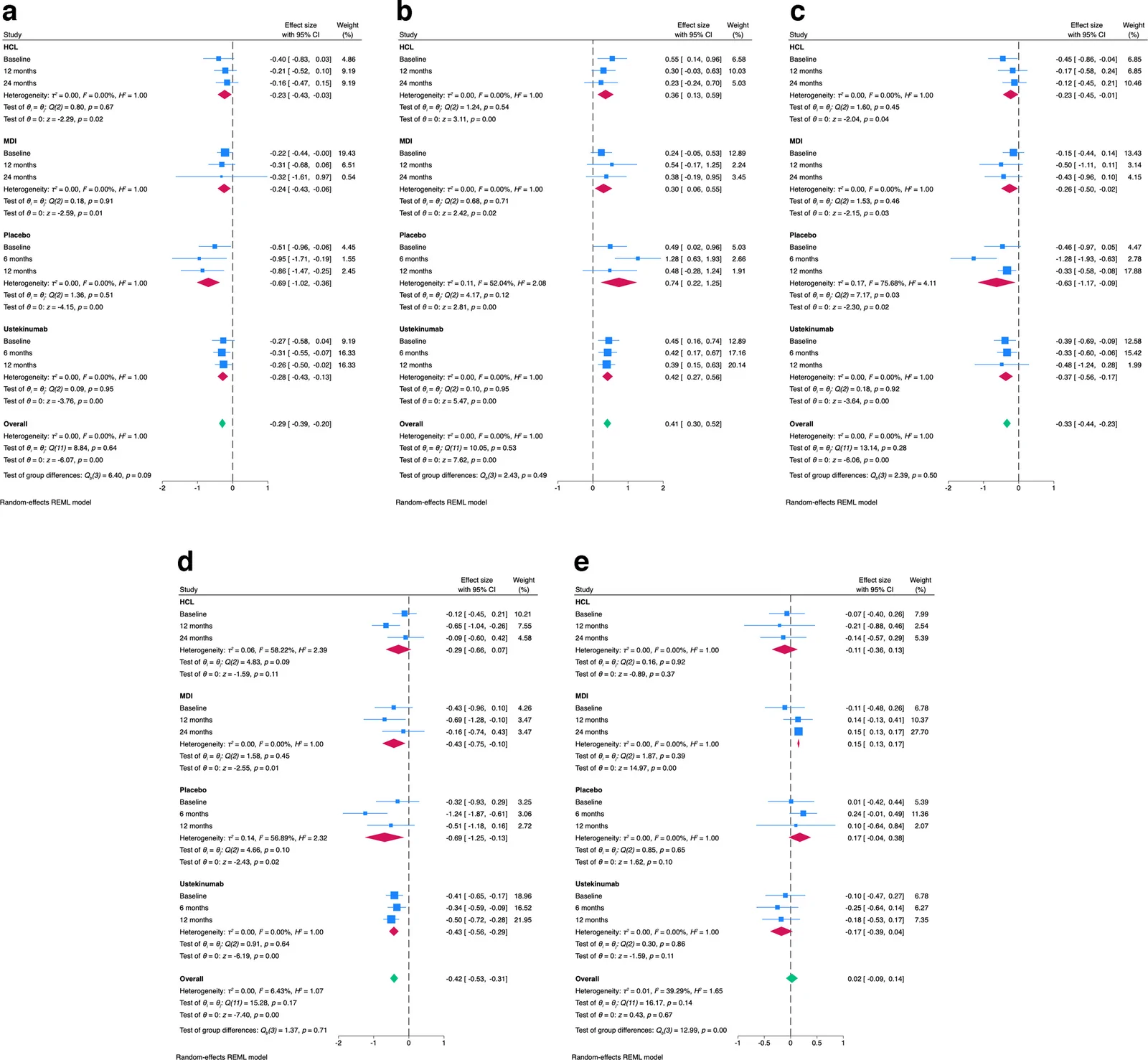
Forest plots showing the association between C-peptide and (**a**) HBA_1c_, (**b**) TIR, (**c**) TAR, (**d**) SD and (**e**) TBR across treatment arms and time points in the CLOuD and USTEKID studies. (**a**) Effect sizes represent standardised regression coefficients (with 95% CIs) for the association between C-peptide and HbA_1c_ at each time point. Negative values indicate that a higher C-peptide is associated with a lower HbA_1c_ value. Size of squares is inversely proportional to the standard error. (**b**) Effect sizes represent standardised regression coefficients (with 95% CIs) for the association between C-peptide and TIR at each time point. Individual study arm effects are shown alongside pooled estimates by treatment type and an overall pooled effect. Positive values indicate that higher C-peptide is associated with more TIR. Size of squares is inversely proportional to the standard error. (**c**) Effect sizes represent standardised regression coefficients (with 95% CIs) for the association between C-peptide and TAR at each time point. Individual study arm effects are shown alongside pooled estimates by treatment type and an overall pooled effect. Negative values indicate that higher C-peptide is associated with less TAR. Size of squares is inversely proportional to the standard error. (**d**) Effect sizes represent standardised regression coefficients (with 95% CIs) for the association between C-peptide and SD at each time point. Individual study arm effects are shown alongside pooled estimates by treatment type and an overall pooled effect Negative values indicate that higher C-peptide is associated with less variability as measured by SD. Size of squares is inversely proportional to the standard error. (**e**) Effect sizes represent standardised regression coefficients (with 95% CIs) for the association between C-peptide and TBR at each time point. Individual study arm effects are shown alongside pooled estimates by treatment type and an overall pooled effect. Neutral values suggest higher C-peptide is not significantly associated with more TBR. Size of squares is inversely proportional to the standard error. CL closed loop; PLC, placebo; REML, restricted maximum likelihood; UST, ustekinumab

A meta-analysis of both trials at all time points identified a clear association between AUC C-peptide levels and HbA_1c_, with higher C-peptide levels being associated with lower HbA_1c_ (β_(std)_=−0.29; 95% CI −0.39, −0.20) (Fig. 2a).

With regard to CGM parameters, meta-analysis revealed higher levels of C-peptide were associated with higher TIR (β_(std)_=0.41; 95% CI 0.30, 0.52), and this effect was observed in all treatment arms including the closed loop arm (Fig. 2b). Higher levels of C-peptide were associated with less time spent above 10.0 mmol/l (TAR) in all treatment arms (β_(std)_=−0.33; 95% CI −0.44, −0.23) (Fig. 2c) and less variability as measured by SD (β_(std)_=−0.42; 95% CI −0.53, −0.31) (Fig. 2d). However, even in the meta-analysis, greater C-peptide was not significantly associated with time below 3.9 mmol/l (TBR) (β_(std)_=0.02; 95% CI −0.09, 0.14) (Fig. 2e).

### Relationship between metabolic parameters and PROMs scores

**Fig. 3:**
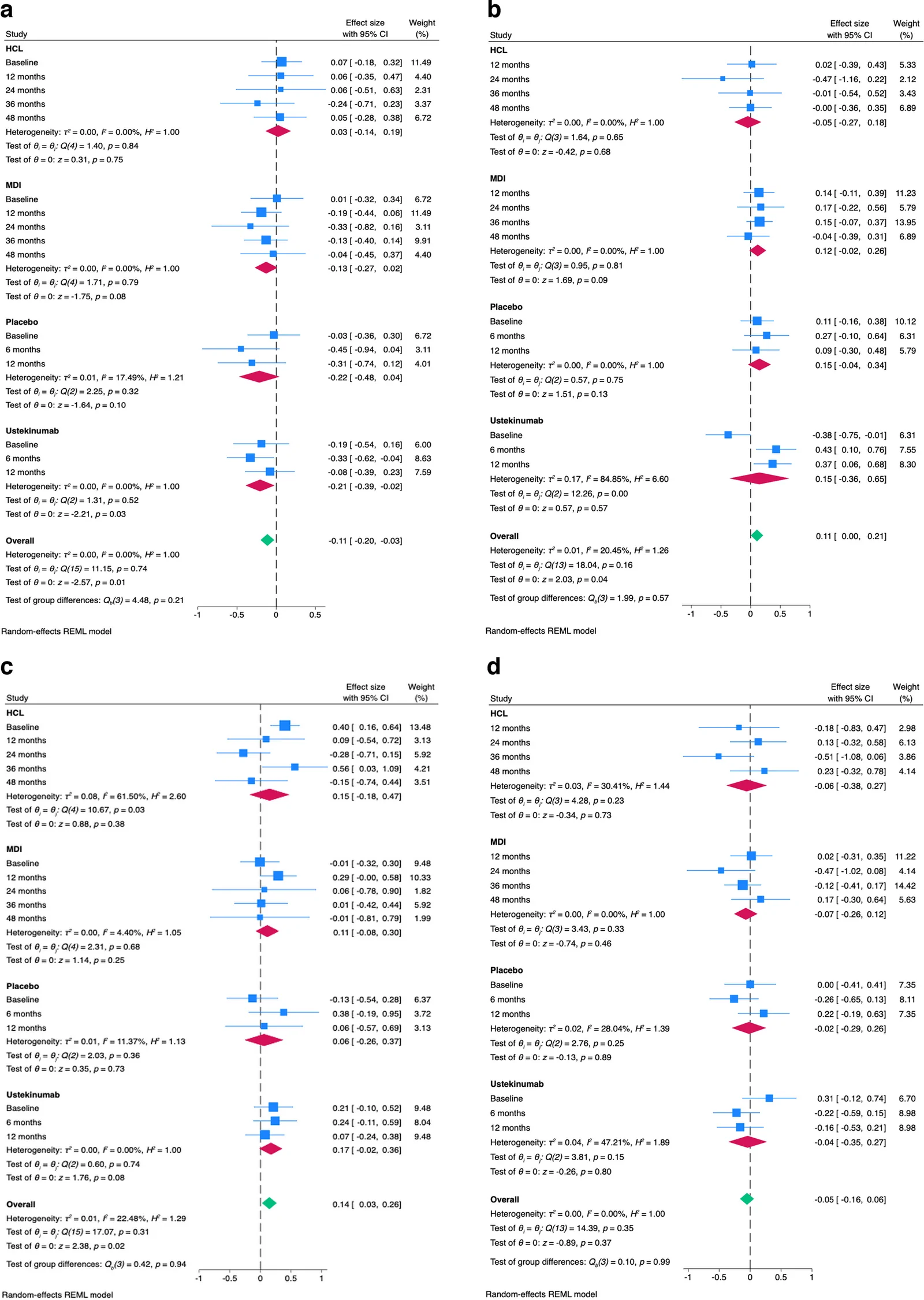
(**a**, **b**) Forest plots showing the association between HbA_1c_ and quality-of-life scores across treatment arms and time points in the CLOuD and USTEKID studies. (**a**) Effect sizes represent standardised regression coefficients (with 95% CIs) for the association between HbA_1c_ and PedsQL at each time point. Negative values indicate that higher HbA_1c_ is associated with lower (worse) PedsQL scores. Size of squares is inversely proportional to the standard error. (**b**) Effect sizes represent standardised regression coefficients (with 95% CIs) for the association between HbA_1c_ and HFS at each time point. Positive values indicate that higher HbA_1c_ is associated with higher (worse) HFS. Size of squares is inversely proportional to the standard error. (**c**, **d**) Forest plots showing the association between TIR and quality-of-life scores across treatment arms and time points in the CLOuD and USTEKID studies. (**c**) Effect sizes represent standardised regression coefficients (with 95% CIs) for the association between TIR and PedsQL at each time point. Positive values indicate that more TIR is associated with higher (better) PedsQL scores. (**d**) Effect sizes represent standardised regression coefficients (with 95% CIs) for the association between TIR and HFS at each time point. Neutral values indicate that more TIR is not significantly associated with lower (better) HFS. REML, restricted maximum likelihood

Meta-analysis of both trials at all time points identified that higher levels of HbA_1c_ were associated with lower (worse) PedsQL scores (β_(std)_=−0.11; 95% CI −0.20, −0.03) (Fig. 3a). The relationship in the group on HCL between HbA_1c_ and PedsQL did not change over time, unlike the other groups. Meta-analysis across all time points revealed that those with higher HbA_1c_ also had higher (worse) HFS scores (β_(std)_=0.11; 95% CI 0.00, 0.21) (Fig. 3b).

**Fig. 4:**
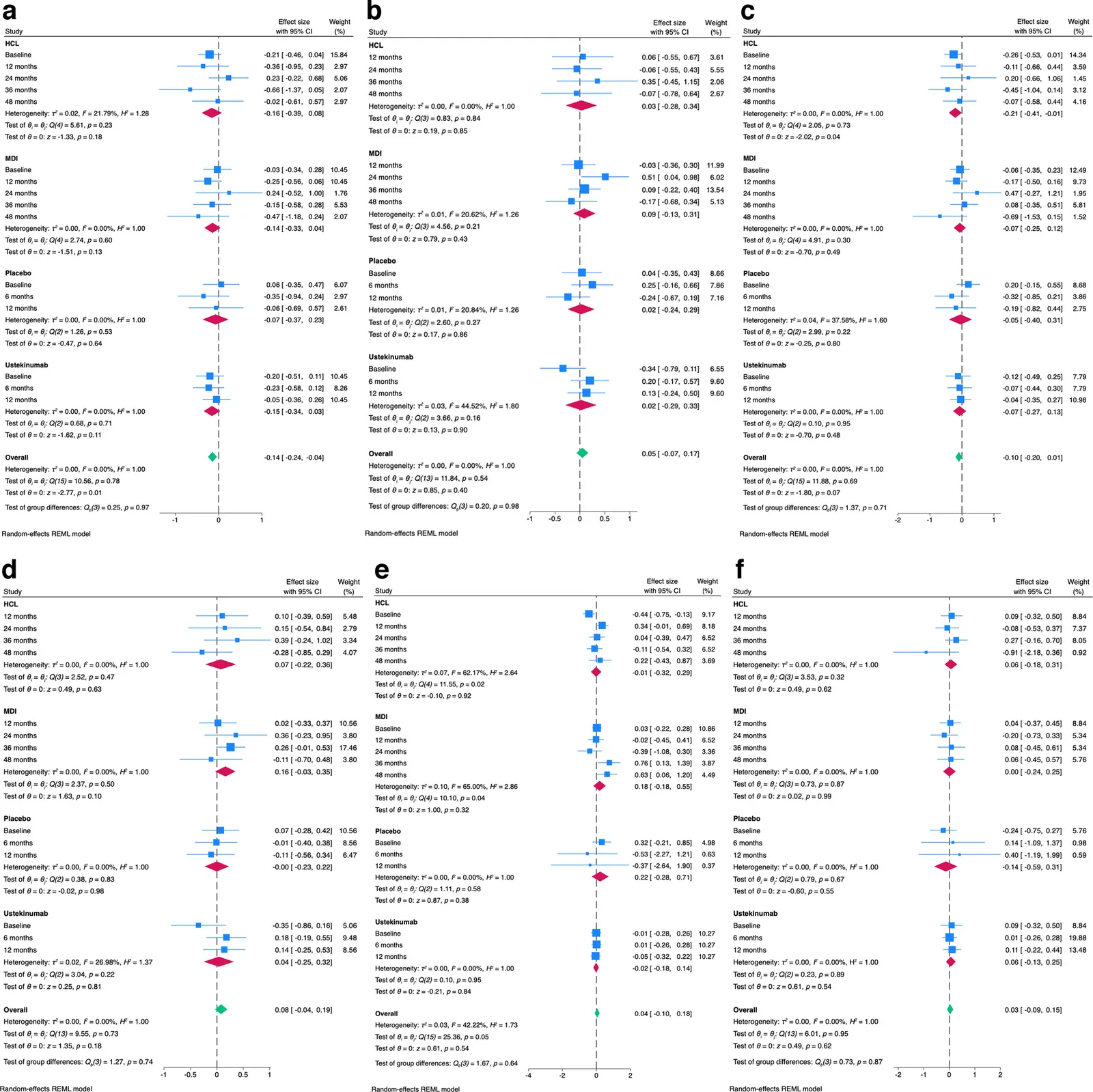
(**a**, **b**) Forest plots showing the association between TAR and quality-of-life scores across treatment arms and time points in the CLOuD and USTEKID studies. (**a**) Effect sizes represent standardised regression coefficients (with 95% CIs) for the association between TAR and PedsQL at each time point. Negative values indicate that more TAR is associated with lower (worse) PedsQL scores. (**b**) Effect sizes represent standardised regression coefficients (with 95% CIs) for the association between TAR and HFS at each time point. Neutral values indicate that higher TAR is not significantly associated with higher (worse) HFS. (**c**, **d**) Forest plots showing the association between variability as shown by SD and quality-of-life scores across treatment arms and time points in the CLOuD and USTEKID studies. (**c**) Effect sizes represent standardised regression coefficients (with 95% CIs) for the association between variability and PedsQL at each time point. Neutral values indicate that more variability is not significantly associated with lower (worse) PedsQL scores. (**d**) Effect sizes represent standardised regression coefficients (with 95% CIs) for the association between variability and HFS at each time point. Neutral values indicate that a greater variability is not significantly associated with higher (worse) HFS. (**e**, **f**) Forest plots showing the association between TBR and quality-of-life scores across treatment arms and time points in the CLOuD and USTEKID studies. (**e**) Effect sizes represent standardised regression coefficients (with 95% CIs) for the association between TBR and PedsQL at each time point. Neutral values indicate that higher TBR is not significantly associated with lower (worse) PedsQL scores. (**f**) Effect sizes represent standardised regression coefficients (with 95% CIs) for the association between TBR and HFS at each time point. Neutral values indicate that higher TBR is not significantly associated with higher (worse) HFS. REML, restricted maximum likelihood

Meta-analysis of both trials at all time points identified that greater TIR was associated with higher PedsQL (β_(std)_=0.14; 95% CI 0.03, 0.26) (Fig. 3c). TAR was also negatively associated with PedsQL (β_(std)_=−0.14; 95% CI −0.24, −0.04) (Fig. 4a). Higher variability as measured by SD showed a trend towards lower PedsQL score but did not reach statistical significance (β_(std)_=−0.10; 95% CI −0.20, 0.01) (Fig. 4c). PedsQL was not associated with TBR (Fig. 4e). HFS was not associated with TIR (β_(std)_=−0.05; 95% CI −0.16, 0.06) or TAR, SD or TBR (Figs 3d, 4b, d, f).

### Relationship between AUC C-peptide and PROMs

**Fig. 5:**
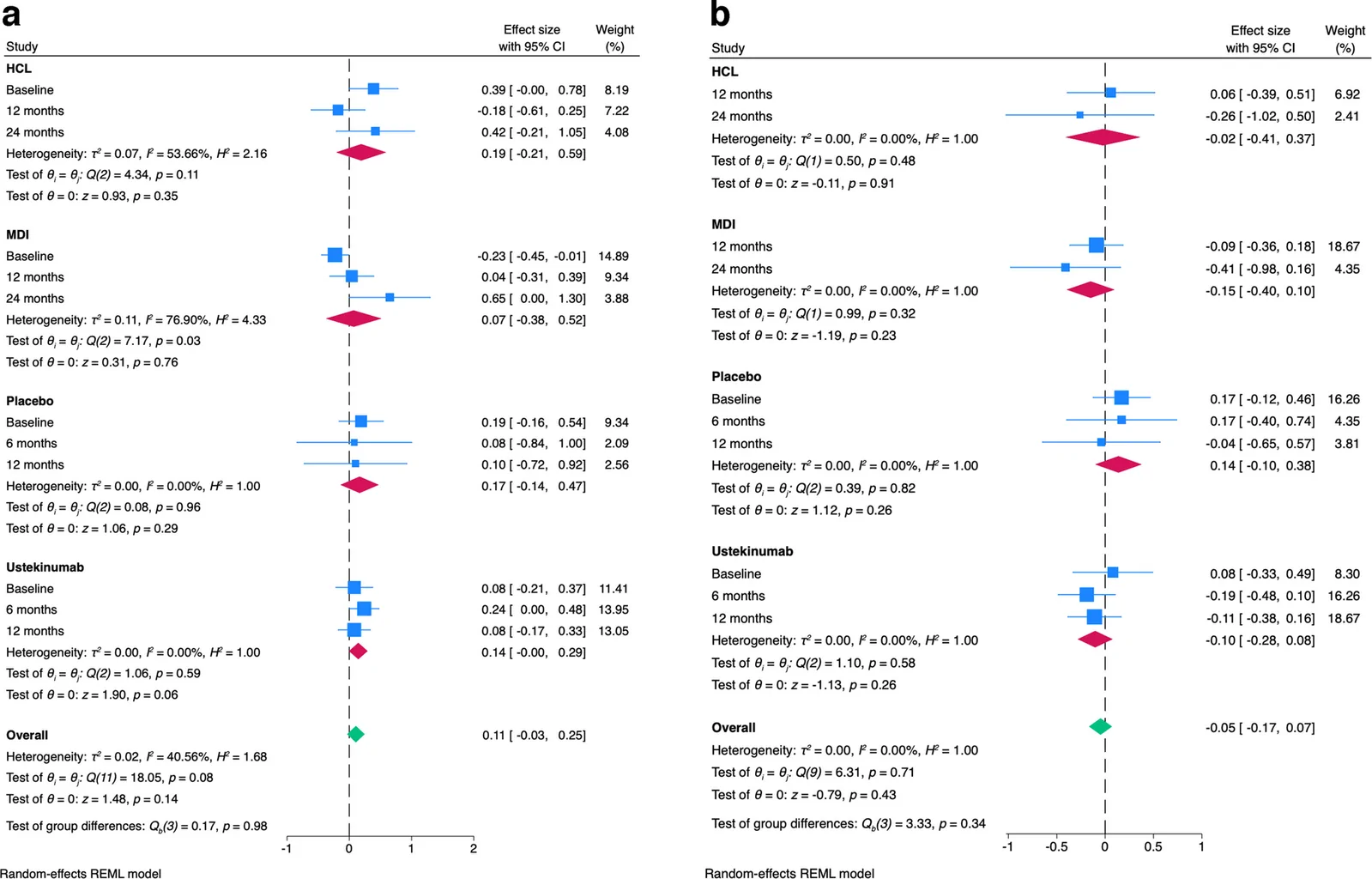
(**a**) Forest plot showing the association between AUC C-peptide (beta cell function) and quality of life (PedsQL scores) across treatment arms and time points in the CLOuD and USTEKID trials. Effect sizes represent standardised regression coefficients (with 95% CIs) for the association between AUC C-peptide and QoL at each time point. Positive values indicate that higher C-peptide is associated with better QoL. Size of squares is inversely proportional to the standard error. (**b**) Forest plot showing the association between C-peptide and hypofear across treatment arms and time points in the CLOuD and USTEKID trials. Effect sizes represent standardised regression coefficients (with 95% CIs) for the association between AUC C-peptide and QoL at each time point. Positive values indicate that higher C-peptide is associated with better QoL. Size of squares is inversely proportional to the standard error. REML, restricted maximum likelihood

Meta-analysis of both trials across all time points identified that although higher AUC C-peptide levels showed a trend towards higher PedsQL scores, this did not reach significance (β_(std)_=0.11; 95% CI −0.03, 0.25) (Fig. 5a). Similarly, while higher AUC C-peptide levels trended towards lower levels of HFS, this also did not reach significance (β_(std)_=−0.05; 95% CI −0.17, 0.07) (Fig. 5b).

## Discussion

In this report, by meta-analysing results across both trials we observed consistent but modest correlations between beta cell function (AUC C-peptide) and metabolic parameters (HbA_1c_, TIR, TAR and SD, but not TBR) (Fig. 2a–e). There was also a clear correlation between several metabolic parameters and quality of life as measured by PedsQL (HbA_1c_, TIR and TAR, but not SD or TBR) (Figs 3a, c, 4a, c, e). Hypofear as measured by HFS correlated positively with HbA_1c_ (high HbA_1c_, worse hypofear) but not with any CGM metrics (including TBR) (Figs 3b, d, 4b, d, f). Beta cell function showed a trend towards improved quality of life and less hypofear but these associations did not reach significance. Taken together, these findings indicate that PROMs as measured by the tools used here are less sensitive to changes in beta cell function than metabolic parameters.

These findings suggest that if existing PROMs are used as endpoints in clinical trials of interventions to preserve beta cell function in new-onset type 1 diabetes, it would be challenging to observe any evidence of association. Theoretically it may be possible to show differences but large sample sizes, far larger than for metabolic outcomes, will be required. Furthermore, it appears that quality of life metrics are more sensitive to differences in beta cell function in the new-onset period than hypofear. Consistent with these observations, no significant impact of treatment with immunotherapy or HCL technology on PedsQL or HFS scores was seen. This is despite USTEKID showing an improvement in C-peptide preservation [22] and CLOuD showing an improvement in HbA_1c_ [23]. It is noteworthy that HbA_1c_ had stronger associations with hypofear (Fig. 3b) than was observed for TIR and hypofear (Fig. 3d); however, both were strongly associated with PedsQL (Fig. 3a, c).

Our findings are consistent with previous evidence demonstrating that preserved C-peptide secretion is associated with improved glycaemic outcomes and CGM metrics such as TIR and glycaemic variability [25, 26]. The PROMs used in CLOuD and USTEKID were developed more than 15 years ago, before the widespread use of CGM and closed loop systems. Recently, new psychometric tools have been created to address the needs of contemporary early-stage type 1 diabetes populations, including the Short Form of the State Anxiety Inventory for Children at Risk for Type 1 Diabetes (SAI-CH-6), which specifically captures diabetes-related anxiety in children in the pre-diagnosis or early disease stages [27]. The development and validation of such instruments will be essential for future immunotherapy and technology trials. Recent studies highlight that while advanced technologies improve glycaemic metrics, they may not reduce the daily psychosocial burden associated with living with diabetes [28–31]. Young people continue to experience challenges such as visible device wear, data overload and persistent self-regulation demands, which are not fully captured by existing quality-of-life tools. This further supports the need for updated PROMs sensitive to the lived experience of modern diabetes therapy [28, 30, 32].

Interestingly, we found no significant change in PedsQL or HFS scores over time, even extending up to 48 months, in CLOuD. It might be expected that individuals become ‘adjusted’ to the diagnosis over time, but we did not observe this. This is important in designing studies to use PROMs as endpoints in the new-onset period. It indicates that while larger sample size may help detect differences in PROMs, longer follow-up is unlikely to be valuable. It is possible that this reflects a deficiency in the tools used, as these were not designed to detect adjustment following diagnosis, as PROMs used in both USTEKID and CLOuD were developed with youths who had mean duration of type 1 diabetes >5 years [7, 8]. There may be an argument for developing PROMs tools specifically to target the new-onset period. However, it was notable that there was a very wide range of PROMs scores between individuals and that baseline scores strongly predicted values at 12 and 48 months of follow-up (ESM Figs. 2 and 3). There was also a strong correlation between parent/carer and participant scores (ESM Figs. 1 and 3). Taken together, this suggests personal and family factors have a strong influence on PROMs scores following diagnosis of type 1 diabetes, which may limit our ability to see any impact of preservation of beta cell function. It is also noteworthy that parents/carers consistently had higher hypofear scores than their children with type 1 diabetes in keeping with an earlier study [33].

Education and structured training at diagnosis may also shape early psychological responses to diabetes and therefore influence PROMs, including HFS scores. Standardised, curriculum-based education in paediatric diabetes services, covering insulin adjustment and hypoglycaemia prevention, was provided in both USTEKID and CLOuD, with additional device-specific training for closed loop users in CLOuD. This early education may help explain the stable PROMs trajectories over time and the generally low levels of hypoglycaemia fear observed.

The observed relationship between metabolic parameters and PROMs warrants further comment. As mentioned in the introduction, this relationship could be causative (i.e. that better metabolic management improves patient well-being), but it may also represent reverse causation (i.e. that less anxious patients are able to focus more on glycaemic outcomes). The observation that HCL is associated with improved QoL [34–36] in other studies is consistent with a causative relationship, but this may not apply in the new-onset period where individuals have no prior experience with which to make a comparison. This is reflected in the observation that HCL was not associated with improved quality of life in CLOuD, despite improving HbA_1c_, although the study is likely underpowered for a PROMs outcome as discussed above. Large prospective studies or multi-study meta-analyses will be required to resolve this. Adjustment for metabolic change in such large datasets may then be able to indicate whether preservation of beta cell function can have a direct impact on PROMs, or whether all of the effect is mediated via improvements in metabolic management.

Strengths of our study include recruitment of participants early after diagnosis, with multiple assessments of different quality-of-life scores, including generic and disease-specific, and with contemporaneous metabolic and beta cell preservation assessments. Limitations of this study include missing data, particularly at later assessments, and generalisability as the people recruited to trials may be from higher educational and lower deprivation backgrounds than the general population with type 1 diabetes. Missing PROMs data, especially at later assessments, represent an important limitation that may have reduced our ability to detect modest relationships between beta cell function and quality-of-life outcomes. Future pooled analyses or meta-studies combining similar datasets may help overcome these power constraints. Data are cross-sectional so potentially subject to confounding and reverse causation. Sex may influence both metabolic outcomes and patient-reported outcomes, including glycaemic management, hypoglycaemia-related fear and psychosocial burden. As this study was not powered for sex-stratified analyses we were unable to explore these effects, which may limit the generalisability of findings by sex. Future studies should incorporate sex- and gender-sensitive analyses to better understand potential differences in the relationships between beta cell function, metabolic control and PROMs.

Another limitation of this study is that although changes were seen across the study arms in AUC C-peptide for ustekinumab [22] and HbA_1c_ for HCL [23], these may have not been large enough to have sufficient power to impact PROMs.

In conclusion, our findings suggest that while existing PROMs may reflect clinically meaningful aspects of living with established type 1 diabetes, their utility as outcome measures in trials of immunotherapy or advanced technologies in the newly diagnosed is limited by their relative insensitivity, due to longitudinal stability and inter-individual variability resulting in limited power to detect an association with beta cell preservation. Both the PedsQL Diabetes Module and the Hypoglycaemia Fear Survey were developed more than 15 years ago, when CGM and HCL systems were not yet in use. As a result, some questionnaire items (e.g. concern about ‘unrecognised hypoglycaemia’ or ‘blood glucose testing difficulties’) may no longer reflect the realities of current care, potentially limiting sensitivity to change in contemporary cohorts. Novel, context-specific PROMs, particularly for early-stage or pre-clinical type 1 diabetes, are probably needed to capture meaningful changes in participant experience in clinical trials of beta cell preservation in new-onset type 1 diabetes. Multi-study meta-analyses may also increase power but will require consensus within the community to ensure consistency in measures used across trials.

## Supplementary Information

Below is the link to the electronic supplementary material.

ESM Figs (PDF 253 KB)

## Data Availability

The datasets analysed during the current study (CLOuD and USTEKID) are available from the corresponding author on reasonable request, subject to trial governance and ethical approvals.

## Abbreviations

CGM: Continuous glucose monitoring
HCL: Hybrid closed loop
HFS: Hypoglycaemia fear survey
HFS-B: Hypoglycaemia fear survey behaviour subscale
HFS-W: Hypoglycaemia fear survey worry subscale
MDI: Multiple daily injections
PedsQL: Paediatric Quality of Life Inventory diabetes module
PROM: Participant-reported outcome measure
TAR: Time above range
TBR: Time below range
TIR: Time in range

## Authors’ relationships and activities

CB is a member of the editorial board of *Diabetologia*. RH reports having received speaker honoraria from Eli Lilly, Dexcom and Novo Nordisk; receiving license fees from BBraun; patents related to closed loop; being a consultant to Abbott Diabetes Care; and being a director at CamDiab. The authors declare that there are no other relationships or activities that might bias, or be perceived to bias, their work.
