# Supplementary material for: Relationship between participant-reported outcomes, residual beta cell function and metabolic parameters in youth with newly diagnosed type 1 diabetes
Source: Diabetologia. 2026 Jul 17;69(10):2759–73. doi: 10.1007/s00125-026-06798-z (PMC13541844; doi:10.1007/s00125-026-06798-z)
Supplement: Supplementary file 1 [file 125_2026_6798_MOESM1_ESM.pdf]

ESM Figure 1 – Diabetes Treatment Satisfaction Questionnaire (DTSQ) scores in patient and carer (child and parent) over time in Ustekid study

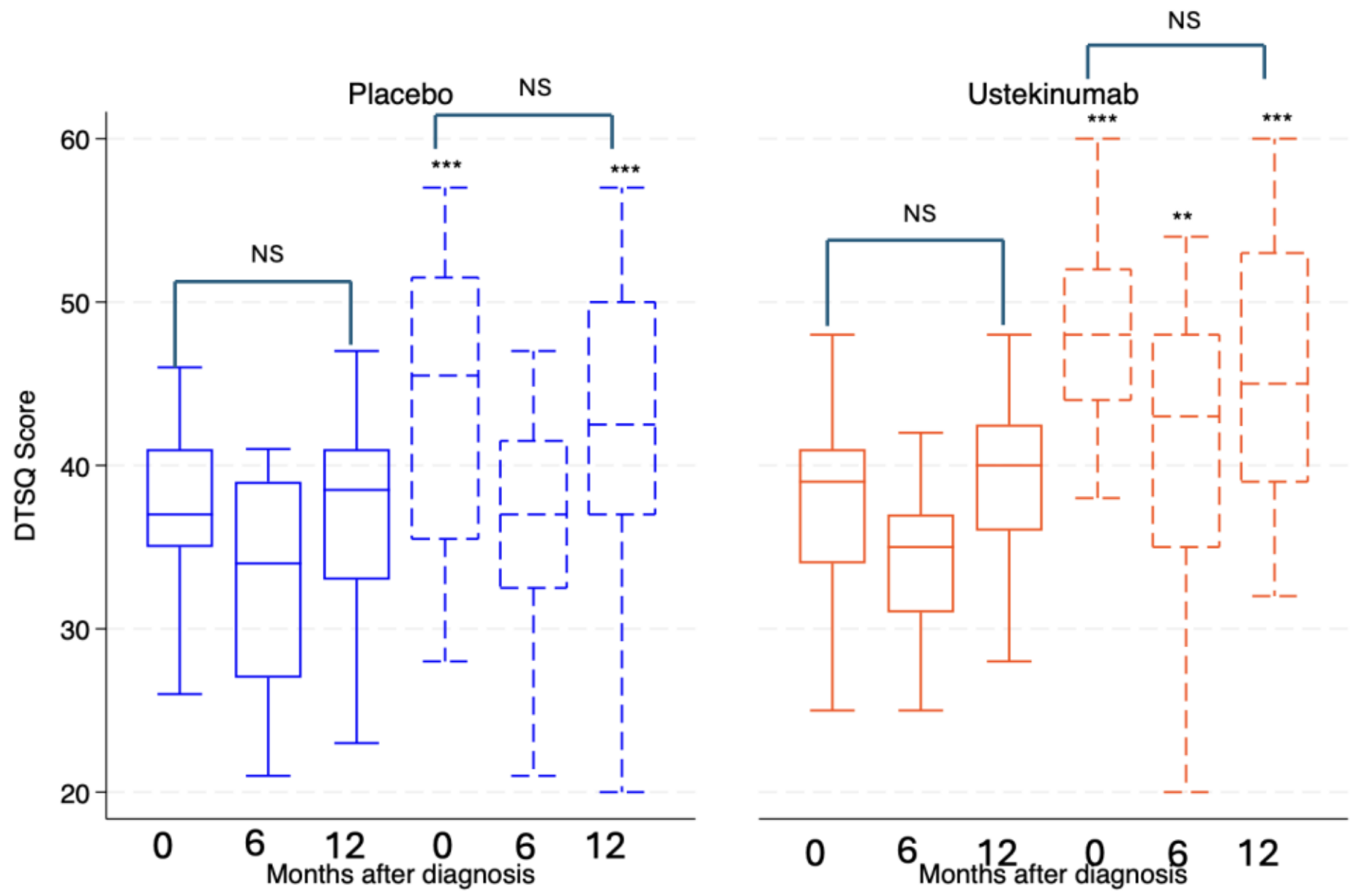

Box plots showing DTSQ scores (median, IQR, 95%) in child (solid line) and in parent (dashed line) at 0,6, and 12 months following diagnosis in placebo participants (blue) and ustekinumab participants (orange). ***p*<0.01, ****p*<0.001 between parent and child scores at this timepoint

ESM Figure 2 – Strengths and Difficulties Questionnaire (SDQ) scores in patient and carer (child and parent) over time in the CLOuD study

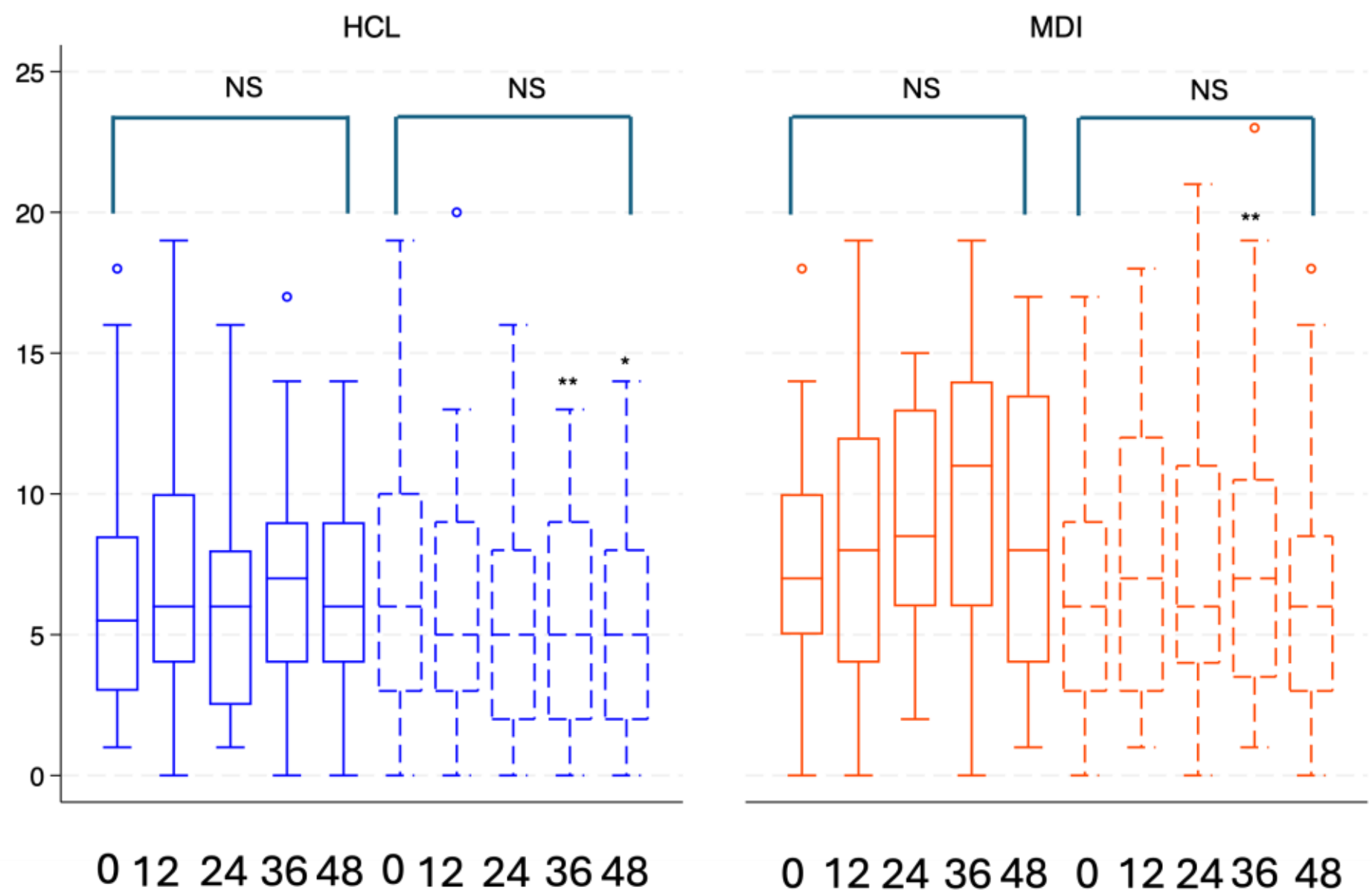

Box plots showing SDQ scores (median, IQR, 95%) in child (solid line) and parent (dashed line) at 0, 12, 24, 36, 48 months following diagnosis in participants receiving HCL (blue) and MDI (orange). ***p*<0.01 between parent and child scores at this timepoint

ESM Figure 3A and 3B – Correlation between baseline and 12, and 48-month PEDSQL scores in participants in USTEKID and CLOuD studies.

A

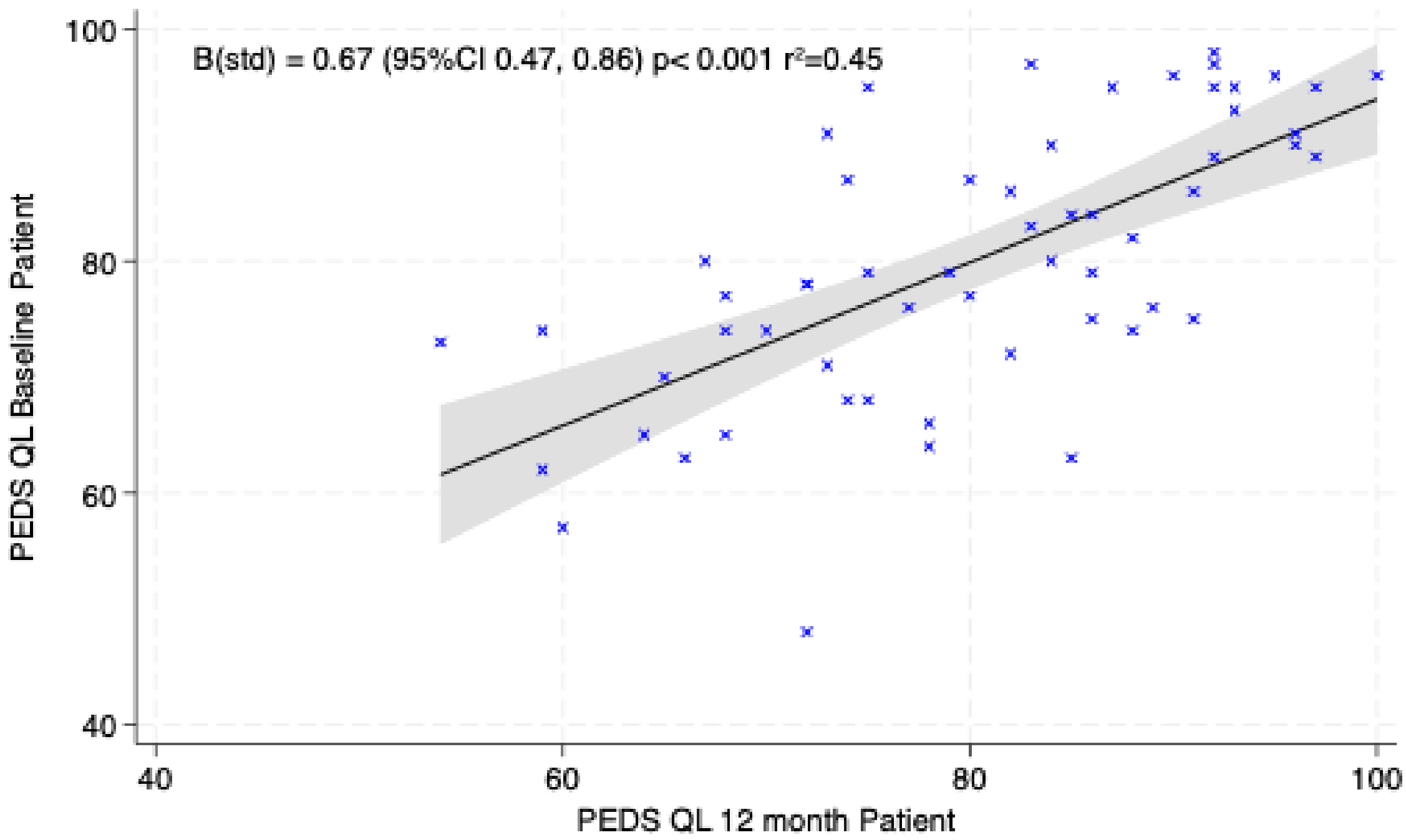

B

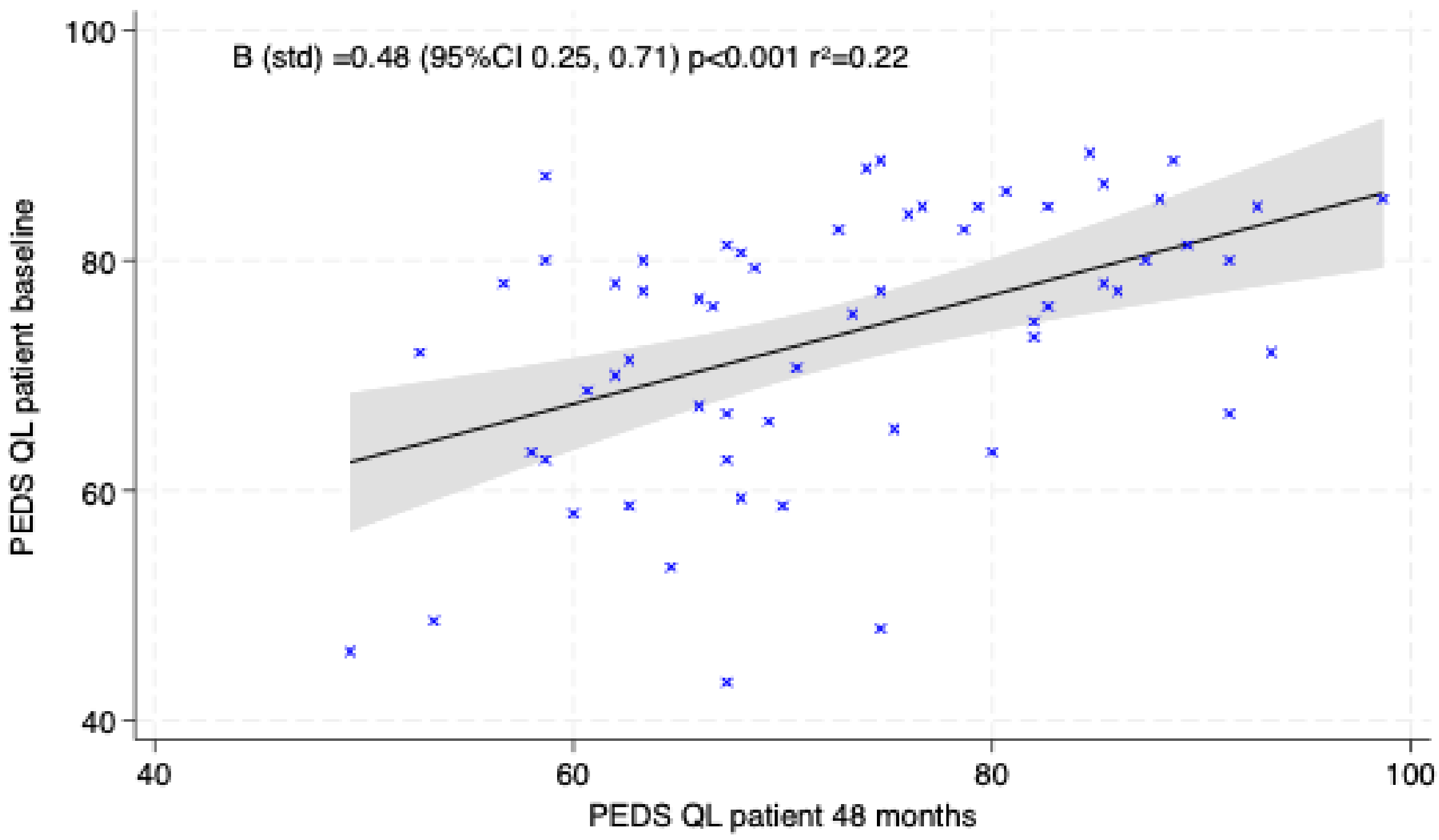

(a) Scatter plot showing the relationship between patient-reported PEDSQL scores at baseline and 12 months post-diagnosis in USTEKID study; (b) Scatter plot showing the relationship between patient-reported PEDSQL scores at baseline and 48 months post-

diagnosis in CLOuD study. The red line represents the line of best fit with 95% confidence interval shown in grey shading.

ESM Figure 3C and 3D – Correlation between parent and child (patient) PEDSQL scores in USTEKID and CLOuD studies.

C

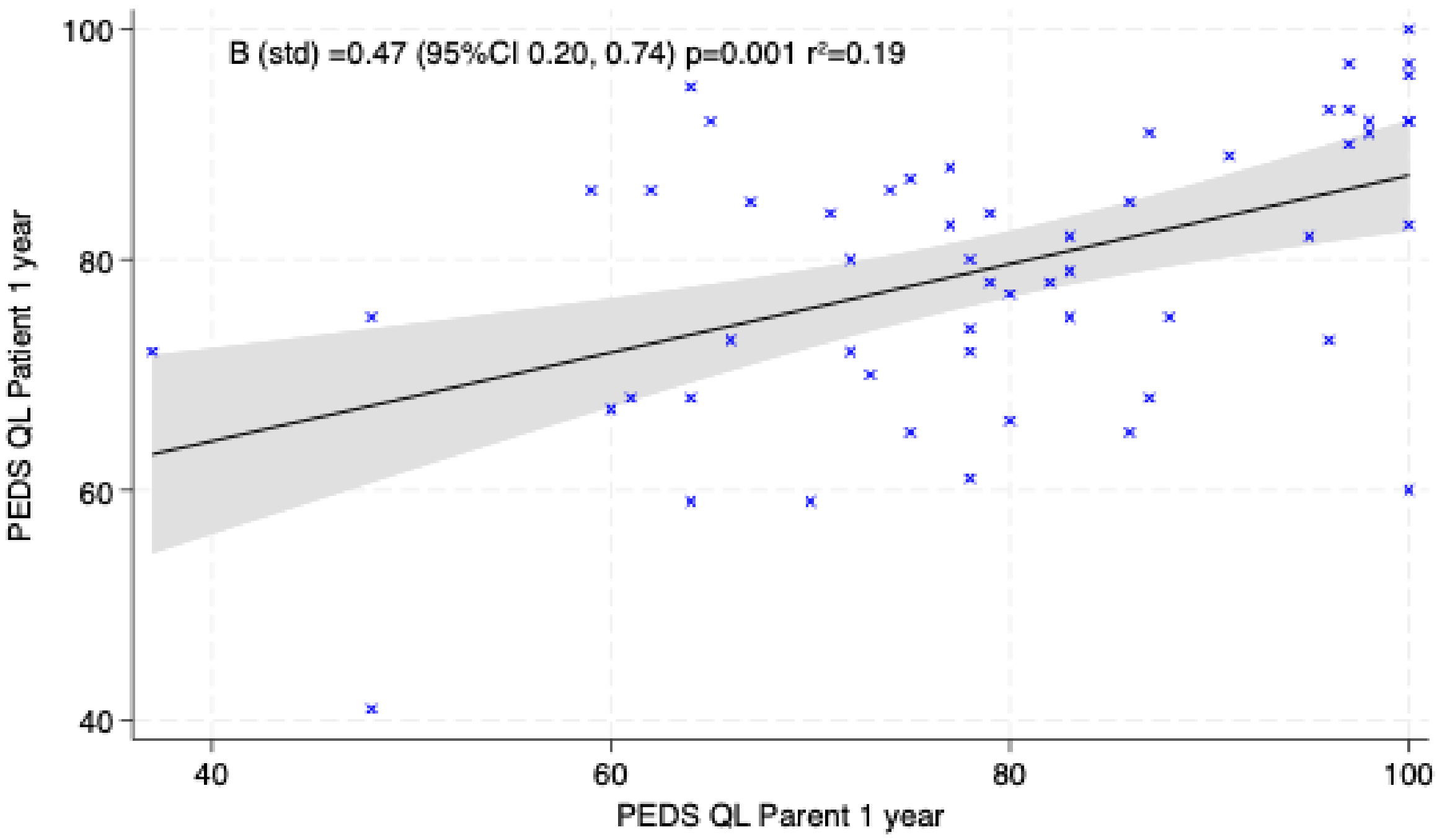

q

D

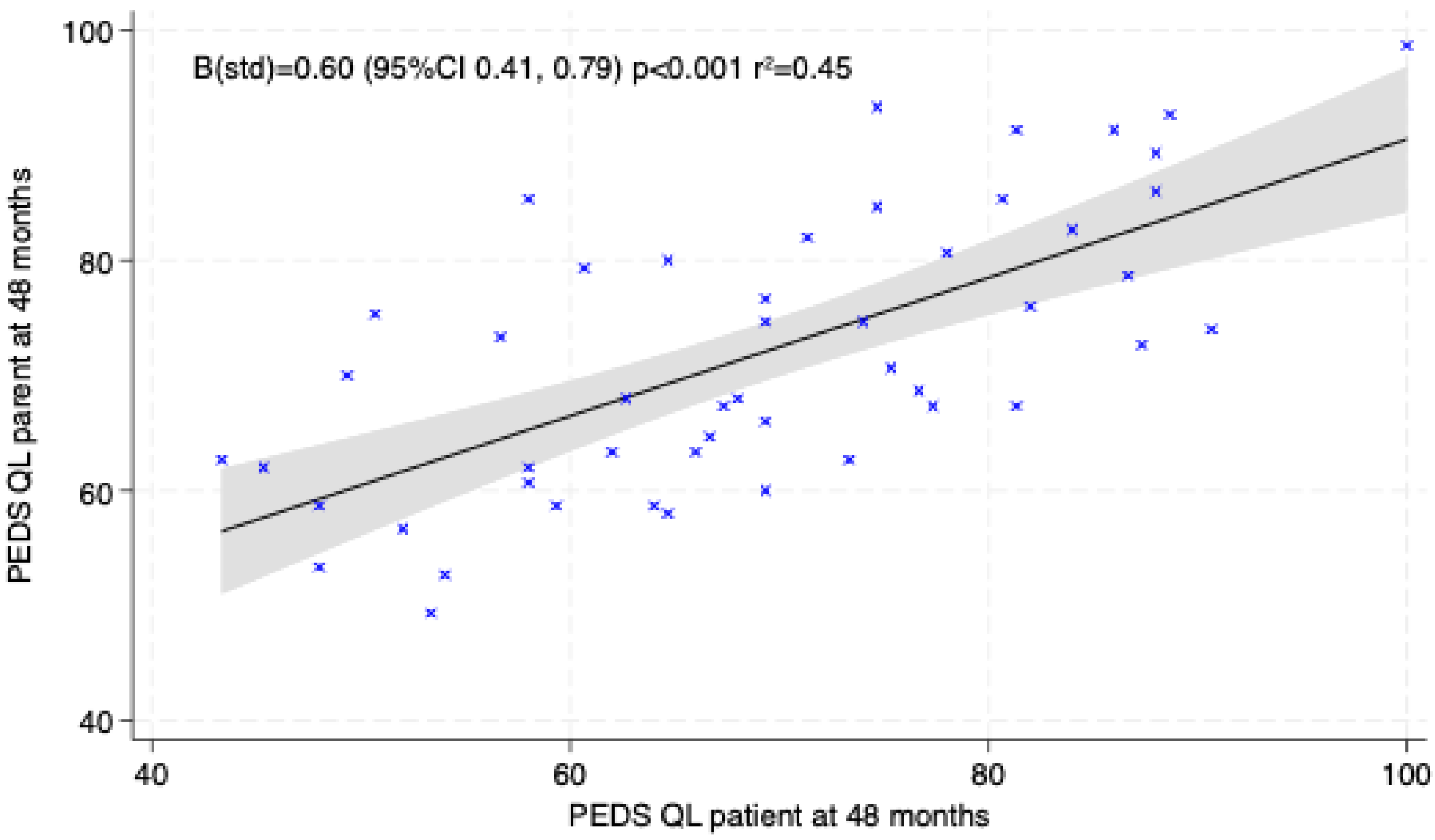

(c) Scatter plot showing the relationship between parent and patient PEDSQL scores at 12 months post-diagnosis in USTEKID study; (d) Scatter plot showing the relationship

between parent and patient PEDSQL scores at 48 months post-diagnosis in CLOuD study. The red line represents the line of best fit with 95% confidence interval shown in grey shading.
